# Mechanisms of KGF Mediated Signaling in Pancreatic Duct Cell Proliferation and Differentiation

**DOI:** 10.1371/journal.pone.0004734

**Published:** 2009-03-06

**Authors:** Benjamin Uzan, Florence Figeac, Bernard Portha, Jamileh Movassat

**Affiliations:** Laboratory of Pathophysiology of Nutrition, Paris Diderot- Paris 7 University, Department of Life Science, CNRS/UMR 7059, Paris, France; University of Bremen, Germany

## Abstract

**Background:**

Keratinocyte growth factor (KGF; palifermin) is a growth factor with a high degree of specificity for epithelial cells. KGF is an important effector of epithelial growth and tissue homeostasis in various organs including the pancreas.

Here we investigated the intracellular signaling pathways involved in the mediation of pancreatic ductal cell proliferation and differentiation induced by exogenous KGF during beta-cell regeneration in diabetic rat.

**Methodology and Results:**

In vitro and in vivo duct cell proliferation was measured by BrdU incorporation assay. The implication of MAPK-ERK1/2 in the mediation of KGF-induced cell proliferation was determined by inactivation of this pathway, using the pharmacological inhibitor or antisense morpholino-oligonucleotides against MEK1. In vivo KGF-induced duct cell differentiation was assessed by the immunolocalization of PDX1 and Glut2 in ductal cells and the implication of PI3K/AKT in this process was investigated.

We showed that KGF exerted a potent mitogenic effect on ductal cells. Both in vitro and in vivo, its effect on cell proliferation was mediated through the activation of ERK1/2 as evidenced by the abolition of duct cell proliferation in the context of MEK/ERK inactivation.

In vivo, KGF treatment triggered ductal cell differentiation as revealed by the expression of PDX1 and Glut2 in a subpopulation of ductal cells via a PI3K-dependent mechanism.

**Conclusion:**

Here we show that KGF promotes beta-cell regeneration by stimulating duct cell proliferation in vivo. Moreover, we demonstrated for the first time that KGF directly induces the expression of PDX1 in some ductal cells thus inducing beta-cell neogenesis. We further explored the molecular mechanisms involved in these processes and showed that the effects of KGF on duct cell proliferation are mediated by the MEK-ERK1/2 pathway, while the KGF-induced cell differentiation is mediated by the PI3K/AKT pathway. These findings might have important implications for the in vivo induction of duct-to-beta cell neogenesis in patients with beta-cell deficiency.

## Introduction

The shortage of islet for transplantation is a major obstacle for the wide application of beta cell replacement therapy in type 1 diabetic patients. To make such a therapy readily available, new sources of insulin-producing cells must be identified. Pancreatic duct cells represent such an alternative source for generation of new beta cells in vitro [1]. Duct compartment has also implications in type 2 diabetes (T2D), since T2D is associated with beta cell loss, and the in situ induction of beta cell neogenesis from ductal progenitor cells may help to restore the beta cell mass in the pancreas of type 2 diabetic patients. Therefore, characterization of stimuli with potential to induce the expansion of duct cells and/or to drive their differentiation into beta cells is of great importance. A variety of growth factors have been described to induce growth and differentiation of ductal cells. Fibroblast growth factors (FGFs) comprise a growing group of structurally related polypeptide mitogens with more than 20 members, most of which stimulate proliferation of a variety of cell types [2], [3]. Keratinocyte growth factor, also called FGF7, is a member of the FGF family. KGF is a paracrine-acting mitogen produced by cells of mesenchymal origin. It acts exclusively through a subset of FGF receptor isoforms (FGFR2IIIb) expressed predominantly by epithelial cells and therefore is a specific mitogen for these cells [4]. Preclinical data from several animal models have demonstrated that recombinant human KGF could enhance the regenerative capacity of epithelial tissues and protect them from a variety of toxic exposures [3]. We and others have previously reported that KGF induces ductal cell proliferation in the pancreas [5]–[8] and promotes beta cell regeneration in neonatal streptozotocin diabetic rats [6]. However the molecular mechanisms underlying the stimulation of beta cell regeneration through the induction of ductal cell proliferation and beta cell neogenesis by KGF are not known.

The present study was undertaken to investigate the intracellular signaling pathways which mediate the growth promoting effects of KGF (palifermin) in pancreatic ducts and to dissect the key steps of duct-to-beta cell differentiation induced by KGF in a model of neonatal diabetes in rat.

In our study, we have established that KGF acts on ductal cells by the the activation of distinct signaling pathways to promote beta cell regeneration. It induces proliferation of the pancreatic ductal cells via the activation of the MEK-ERK1/2 pathway and it triggers duct-to-beta cell differentiation directly by the induction of PDX1 expression in the neonatal ducts via the activation of PI3K/AKT pathway. The duality of the effects of KGF in a different subsets of a unique cell type is an original finding and could have important implication in the potential use of this factor as differentiation promoting agent for regenerative therapies of diabetes.

## Materials and Methods

### Reagents

#### The following chemicals and reagents were used in this study

DMEM was purchased from Promochem (Molsheim, France), penicillin–streptomycin, and trypsin–EDTA solutions from Invitrogen (Cergy Pontoise, France). Fetal bovine serum (FBS) was purchased from Abcys (Paris, France). Keratinocyte growth factor, palifermin (KGF) was generously provided by AMGEN (Thousand Oaks, CA, USA). The pharmacological MEK1 inhibitor, PD98059 and total-ERK1/2 antibody were purchased from Promega (Charbonnières, France). The phospho-ERK1/2 (Thr202/Tyr204) and MEK1 antibodies were purchased from Ozyme (St Quentin en Yvelines, France), cyclin D1 antibody was from Microm (Francheville, France), cyclin D2 antibody and the protein assay reagents were purchased from Interchim (Montluçon, France), actin antibody and wortmannin from Sigma (L'Isle-d'Abeau Chesnes, France), PDX1 antibody from Millipore (St Quentin en Yvelines, France), insulin antibody from MP Biomedicals (Aurora, Ohio, USA), CK 20 antibody from Abcam (Paris, France) and Glut 2 antibody from AbD Serotec (Düsseldorf, Germany). Antisense morpholino-oligonucleotides to knock down endogenous MEK1 (MEK1-AS) expression were designed and produced by Gene Tools LLC (Philomath, Oregon, USA).

### Animals

Wistar rats were fed *ad libitum* with pelleted chow (diet 113, SAVE, Villemoisson-sur-Orge, France). Females were caged with males for one night and pregnancy was detected by abdominal palpation after 14 days. Natural birth occurred 22 days after mating.

All animal experiments were conducted with the approval of French Center for Scientific Research.

### Eight experimental groups were studied

#### STZ/saline group

At the day of birth, rats of this group received an injection of 100 µg/g body weight of streptozotocin (STZ) (Sigma) freshly dissolved in citrate buffer (0.05 M, pH 4.5). From day 2 to day 6, rats received an i.p. injection of saline solution. Rats of this group were sacrificed at day 2, 4 or 7 after birth.

#### STZ/KGF group

At the day of birth, rats of this group received a single daily i.p. injection of 100 µg/g body weight of STZ freshly dissolved in citrate buffer (0.05 M, pH 4.5). From day 2 to day 6, rats received an s.c. injection of KGF (palifermin) provided by AMGEN (Thousand Oaks, CA, USA) at the dose of 3 mg/kg body weight. For the evaluation of ERK1/2 activation by KGF, 2-day-old pups received an i.p. injection of KGF 20 minutes before sacrifice.

#### STZ/MEK1-AS group

As for the above groups, Wistar newborns received a single daily i.p injection of STZ. Eight hours after STZ administration rats received a subcutaneous injection of antisense morpholino-oligonucleotides targeted to the MEK1 mRNA translation site (MEK1-AS, 1 nmole/g). On day 2, 8 h before sacrifice, rats underwent another s.c. injection of MEK1-AS.

#### STZ/Std group

Eight hours after STZ administration, rats of STZ group received a subcutaneous injection of non specific standard morpholino-oligonucleotides (Std). On day 2, rats underwent another s.c. injection of Std 8 hours before sacrifice.

#### STZ/MEK1-AS/KGF group

In this group, 2-day-old STZ/MEK1-AS treated animals received an injection of KGF (3 mg/kg body weight) 8 hours before sacrifice.

#### STZ/Std/KGF group

In this group, STZ/Std treated animals received an injection of KGF (3 mg/kg body weight; i.p) 8 hours before sacrifice.

#### STZ/Wortmannin group

Rats of this group were given a subcutaneous injection of wortmannin (0.75 mg/kg body weight), on day 2, 24 hours after the administration of STZ.

#### STZ/Wortmannin/KGF group

In this group, rats were pre-treated with wortmannin for one hour on day 2 and then received an s.c injection of KGF. They were sacrificed 8 hours after KGF administration.

In each group, the pups were left with the dams and kept in an environment of constant temperature, humidity, and day–night cycle. The number of animals per litter was kept at eight. For each experimental group the pups belonged to at least three different litters. All the neonates were tested for blood glucose concentration (Glucotrend, Roche) 24 h after STZ administration. Animals were included in the study only if their glycemia was higher than 9.35 mmol/l.

Animals were killed on day 2, 4 or 7 after birth. One hour before the sacrifice, animals were given an injection of 5′-bromo-2′-deoxyuridine (BrdU) (Sigma) at the dose of 50 mg/kg, i.p.

Pancreases were removed and half of the tails and heads were used for immunohistochemistry and the other halves were used for western blot analysis.

### Immunohistochemistry

Pancreases were fixed in aqueous Bouin's solution for 24 h and embedded in Paraplast. Each piece of pancreatic tissue was serially sectioned (5 µm) throughout its length. Sections at a fixed interval throughout the block (every 35^th^ section) were immunostained for BrdU using the cell proliferation kit (GE Healthcare, Orsay, France) as described previously [9]. To identify ductal cells, pancreatic sections were stained for cytokeratin 20 (CK20), a specific marker of ductal cells in rat [10], [11].

To estimate the ductal cell replication rate, ductal cells were counted in CK20/BrdU double- stained pancreatic sections. After BrdU staining, sections were stained for CK20 using an alkaline phosphatase-conjugated antibody as secondary antibody. Results were expressed as the percentage of BrdU-positive ductal cells. At least 800 ductal cells were counted per pancreas, and four different sections were analyzed for each pancreas.

In order to assess ductal cell differentiation, we sought to identify ductal cells with intermediate phenotype. These consisted of cells that expressed specific beta cell markers that are not normally expressed in ductal cells. As such markers, we investigated the expression of PDX1 and the beta cell specific glucose transporter, Glut 2. Since both CK20 and PDX1 antibodies were raised in rabbit, double staining using both antibodies on the same section could not be carried out. Therefore, in order to localize the PDX1 expressing ductal cells in the pancreatic sections of 2-day-old rats , double staining for insulin and PDX1 were performed in sections adjacent to those that were stained for CK20. To estimate the number of PDX1-positive cells, ductal cells that were positive for PDX1, but negative for insulin were counted and the results were expressed as the percentage of PDX1-positive/insulin-negative ductal cells over total ductal cells. For all in vivo studies at least 4 animals were used in each group. For the identification of Glut 2 expressing ductal cells, we performed double immunofluorescent staining for Glut 2 and insulin on pancreatic sections of 4-day-old rats. Sections adjacent to these double-stained sections were stained for CK20.

### Cell culture

Human duct cell line Panc-1 used in this study was purchased from ATCC (LCG Promochem Molsheim, France). Panc-1 cells were grown in DMEM supplemented with 10% FBS and penicillin–streptomycin (1%) in a humidified atmosphere of 95% O_2_, 5% CO_2_ at 37°C. The cells were regularly seeded into T75 flask (VWR International, Fontenay-sous-Bois, France). Media were changed twice weekly.

### Inhibition of MAP kinases ERK1/2 pathway

ERK1/2 pathway was inactivated by the inhibition of its specific upstream activator MEK1. MEK1 inhibition was performed both by using pharmacological inhibitor or by the inhibition of its translation by the means of specific antisense oligonucleotides.

Cells were seeded in twelve-well plates and after 6 h culture with DMEM supplemented with 10% FBS, cells were changed to serum-free medium for 3 days, followed by appropriate treatments described below.

Pharmacological inhibition of MEK1 was achieved by the use of specific inhibitor PD98059. Different doses (1, 10 and 20 µM) of PD98059 were tested. For pharmacological inhibition of MEK1, cells were pre-treated with the inhibitor 1 h before KGF treatment. Twenty four hours after addition of KGF, cells were processed for western blot analysis or for proliferation assay.

To inhibit MEK1 translation, MEK1 antisense morpholino-oligonucleotides (Gene Tools LLC) (5′-TGGGCGTCGGCTTCTTCTTGGGCAT-3′) were designed based upon the published rat and human common regions of MEK1 cDNA sequence [GenBankTM accession number NM 002755 (human) and NM 001008375 (rat)]. Antisense oligonucleotides were delivered using the DMSO endoporter delivery system as recommended by the manufacturer (Gene Tools LLC). Standard non-specific morpholinos (5′-CCTCTTACCTCAGTTACAATTTATA-3′) were used as control.

Cells were incubated with MEK1 antisense morpholino oligonucleotides or with standard oligonucleotides (Std) for 24 h and then supplemented with KGF (30 ng/ml) for another 24 h. One hour before the end of the experiments, BrdU (10 µM) was added to the culture media. Incubations were terminated by the addition of lysis buffer for western blot analysis or by fixation in 4% formaldehyde for immunocytochemical analysis.

### Western Blotting

Western blot analysis was performed to study phospho- and total-ERK1/2, MEK1, cyclin D1 and cyclin D2 protein expression. Briefly, 10 µg of protein were subjected to SDS-PAGE (10% acrylamide gel) and then transferred to a PVDF membrane for 2 h (120 V) using a Bio-Rad Mini Trans Blot electrophoretic transfer unit (Bio-Rad, Marnes-la-Coquette, France). The membranes were blocked for nonspecific binding with 5% nonfat dry milk in Tris-buffered saline (TBS, 20 mM Tris-HCl, 150 mM NaCl, pH 7.4) supplemented with 0.05% Tween 20 (TTBS) and then probed with the specific primary antibodies. After 3 washes with TTBS, membranes were incubated with appropriate horseradish peroxidase-conjugated secondary antibodies. Separated proteins were visualized by an ECL kit (GE Healthcare) and light emission was captured on X-ray film (GE Healthcare). Intensities of the respective bands were examined by densitometric analysis (Scion Image Analyst program).

### Proliferation assay

Proliferation assay was performed with BrdU incorporation method on Panc-1 cells using a Cell Proliferation Kit (GE Healthcare). BrdU was added to the culture media 1 h before the end of the experiments. Cells were fixed in 4% formaldehyde for 20 min then washed with PBS. Panc-1 cells were then stained for BrdU according to manufacturer's instructions. Cells were then counterstained with hematoxylin.

### Statistical analysis

Data are expressed as the mean values ±sem. Comparisons between the groups were performed by ANOVA and post-hoc (Fisher) or Mann-Withney tests. *p*<0.05 was considered as significant.

## Results

### I- in vitro studies

#### Stimulation of duct cell proliferation by KGF

**Figure 1:**
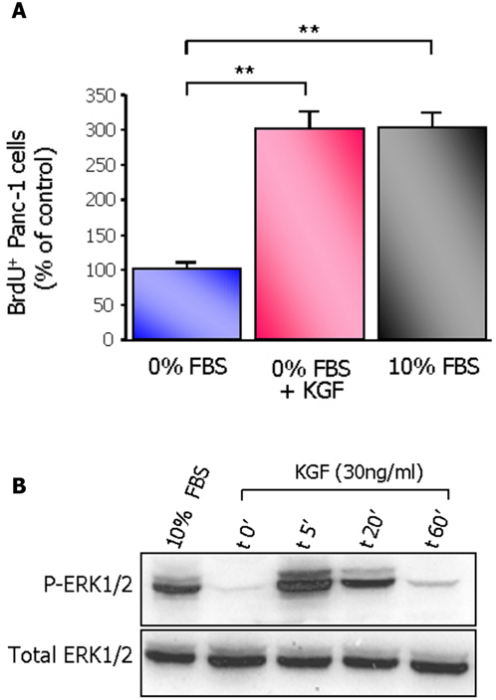
KGF stimulates cell proliferation and activates ERK1/2 pathway. (A) Panc-1 cells were cultured in serum-free medium with or without KGF (30 ng/ml) or with 10% FBS for 24 h. BrdU was added to the culture media 1 h before the end of the experiments. Results are expressed as mean values ±sem of three independent experiments. ** *p*<0.01. (B) Phosphorylation of ERK1/2 was evaluated by western blot analysis in cell extracts from Panc-1 cells stimulated for 5, 20 and 60 minutes with KGF, or with 10% FBS for 20 minutes. Western blot analysis of total-ERK1/2 protein was used as control.

Panc-1 cells have been rendered quiescent by 72 h serum deprivation (control group) and were then treated with KGF or 10% FBS for 24 h. The exposure to KGF potently stimulated the mitotic activity of the cells (Figure 1A) as evidence by the 200% increase of the rate of cell proliferation in those cells as compared to that of the control group. The stimulation of proliferation induced by KGF was similar to that measured in cells treated with 10% FBS (Figure 1A).

#### Activation of ERK1/2 pathway by KGF

The time-course analysis of ERK1/2 phosphorylation in cells treated with KGF showed a prompt activation of ERK1/2 which peaked at 5 min after stimulation. The activation of ERK1/2 was transient and the basal levels of ERK1/2 phosphorylation were recovered 1 h after stimulation (Figure 1B).

At the time-point 20 min after stimulation, KGF-induced activation of ERK1/2 resembled that induced by 10% FBS.

#### Inactivation of ERK1/2 pathway by MEK1 inhibitors and its effects on KGF-induced cell proliferation

**Figure 2:**
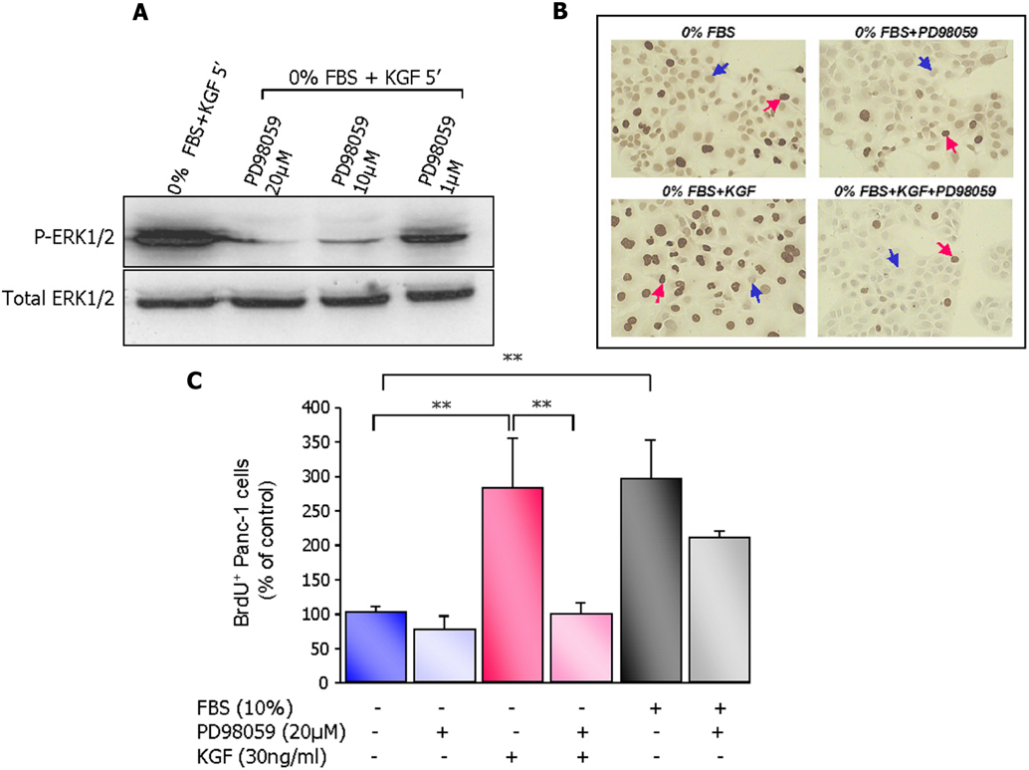
Inactivation of ERK1/2 pathway by pharmacological inhibitor abolishes KGF-induced Panc-1 cell proliferation. (A) Panc-1 cells were pre-incubated for 1 h with 20 µM, 10 µM and 1 µM of PD98059, an inhibitor of MEK1. Cells were then treated for 5 minutes with 30 ng/ml KGF. Cell lysates were analyzed by western blot using specific antibodies against P-ERK1/2 and total-ERK1/2. (B) Panc-1 cells were cultured in serum-free medium. Cells were then exposed or not to KGF or PD98059 alone, or to the combination of the two agents for 24 h. Cell proliferation was assessed by BrdU staining (brown nuclei). Red arrows show BrdU-positive Panc-1 cells and blue arrows show BrdU-negative Panc-1 cells (actual magnification ×250). (C) Panc-1 cells were treated or not with 20 µM PD98059, 1 h prior to the 24 h incubation with KGF or 10% FBS. BrdU labelling index of Panc-1 cells was evaluated and expressed as the percentage of BrdU-positive Panc-1 cells. Results are presented as mean values ±sem of three independent experiments. ** *p*<0.01.

To establish the functional link between ERK1/2 activation and the stimulation of cell proliferation induced by KGF, we proceeded to the inactivation of the only known upstream activator of ERK1/2 proteins. MEK1 was first inactivated by its pharmacological inhibitor PD98059. KGF-induced proliferation of Panc-1 cells assessed by BrdU staining (Figure 2B) was completely abolished by blockade of ERK1/2 phosphorylation caused by PD98059 (Figures 2A and 2C). In contrast, MEK1 inactivation and the consequent inhibition of ERK1/2 pathway, caused only a slight and non significant decrease in the rate of cell proliferation induced by 10% FBS (Figure 2C).

**Figure 3:**
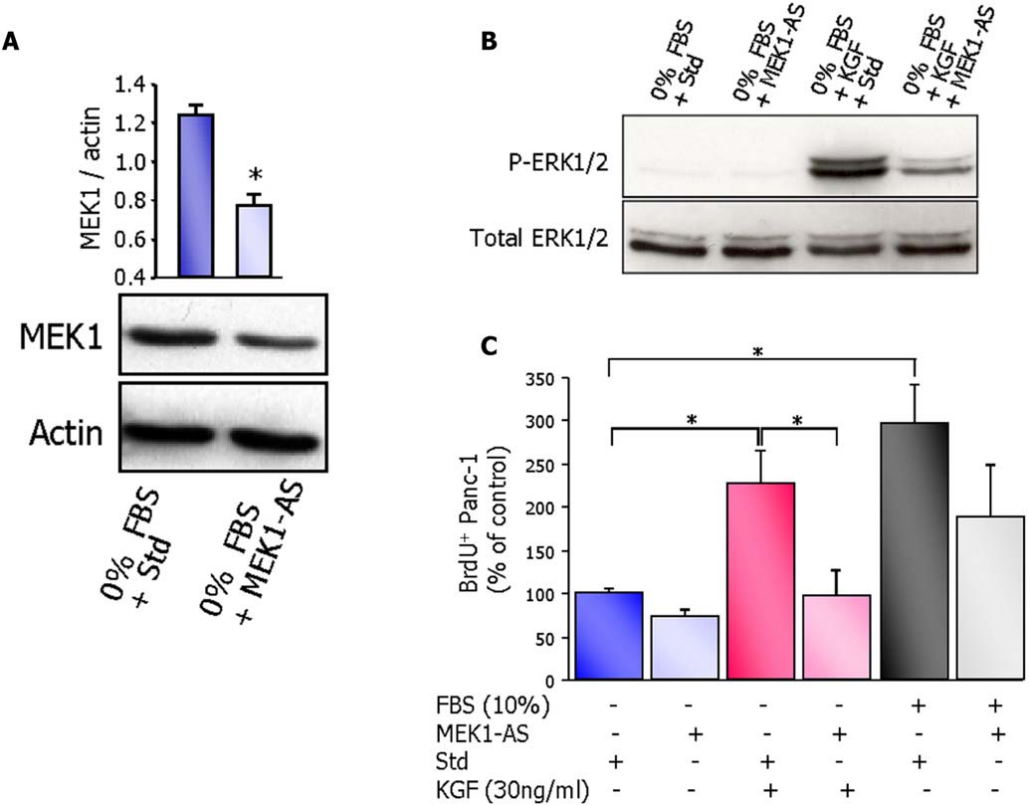
Inactivation of ERK1/2 pathway by antisense morpholino-oligonucleotides abolishes KGF-induced proliferation of Panc-1 cells. (A) Panc-1 cells were cultured in serum-free medium and treated for 24 h with MEK1 antisense morpholino-oligonucleotides (MEK1-AS, 10 nmol/ml) or standard morpholino-oligonucleotides (Std, 10 nmol/ml). Cell lysates were analyzed by western blot using specific antibody against MEK1. Representative blots and MEK1 protein quantification are shown in Figure 3A. Results are expressed as mean values ±sem of 4 independent experiments. * *p*<0.05. (B) Panc-1 cells were treated with MEK1-AS or Std during 24 h, then stimulated with KGF for 5 minutes before the end of the experiments. Cell lysates were analyzed by western blot for P-ERK1/2 and total-ERK1/2 expression. (C) Panc-1 cells were cultured in serum-free medium and pre-treated with MEK1-AS during 24 h prior the addition of KGF or 10% FBS. BrdU labelling index of Panc-1 cells was evaluated and expressed as the percentage of BrdU-positive Panc-1 cells. Results are presented as mean values ±sem of three independent experiments. * *p*<0.05.

Our results from experiments with pharmacological MEK1 inhibitor clearly designate MEK1-ERK1/2 pathway as the main pathway involved in the transduction of KGF biological actions on cell proliferation. However, because of the fundamental interconnectedness of signaling networks, it cannot be ruled-out that interactions with proteins beyond the one intended could be caused by small kinase inhibitors [12], [13]. To definitely exclude the possibility of non-specific effects of PD98059, we used a specific approach to knock down MEK1 protein by antisense morpholino-oligonucleotides. Morpholino-oligonucleotides are useful tools to silence gene expression post-transcriptionally [14], [15]. We first verified that morpholino-oligonucleotides successfully penetrated into the cells by the use of FITC-conjugated MEK1-AS (not shown). Then we showed that the expression of MEK1 protein was decreased by about 40% in Panc-1 cells treated with MEK1-AS (Figure 3A).

Downregulation of MEK1 by specific antisense oligonucleotides blunted ERK1/2 phosphorylation (Figures 3B). KGF-induced cell proliferation was completely abolished in Panc-1 cells treated with MEK1-AS (Figures 3C).

#### Induction of cell cycle regulators by KGF

**Figure 4:**
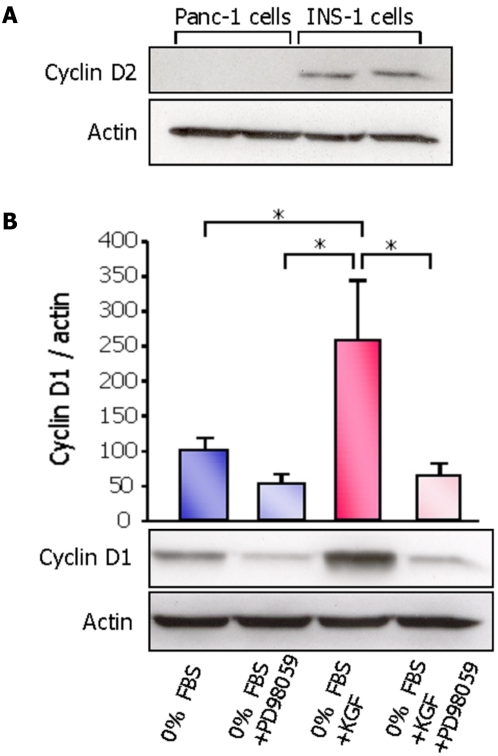
Induction of D-type cyclins by KGF. Cell lysates were analyzed by western blot for cyclin D1 and cyclin D2 expression. (A) Cells were cultured in medium with 10% FBS. Basal levels of cyclin D2 were not detectable in Panc-1 cells as compared to its expression in insulinoma cell line, INS-1 used as control for the specificity of antibody. (B) Panc-1 cells were cultured in serum-free medium. Cells were then exposed or not to KGF or PD98059 alone, or to the combination of the two agents for 8 h. Cell lysates were analyzed by western blot for cyclin D1 expression. Results are expressed as mean values ±sem of three independent experiments. * *p*<0.05.

We evaluated the expression of D-type cyclins (D1 and D2) in Panc-1 cells by western blot analysis. Cyclin D2 was not expressed in Panc-1 cells (Figure 4A) whereas cyclin D1 was expressed at a low level in serum deprived Panc-1 cells. We analyzed the effect of KGF on the expression of cyclin D1, 8 h after incubation with the peptide. KGF significantly increased the levels of cyclin D1 protein in the cells (Figure 4B). Blockade of ERK1/2 pathway by PD98059 silenced the KGF-induced expression of cyclin D1 indicating that induction of this protein by KGF is ERK1/2 dependent (Figure 4B).

### II- In vivo studies

#### Activation of ERK1/2 pathway by KGF

**Figure 5:**
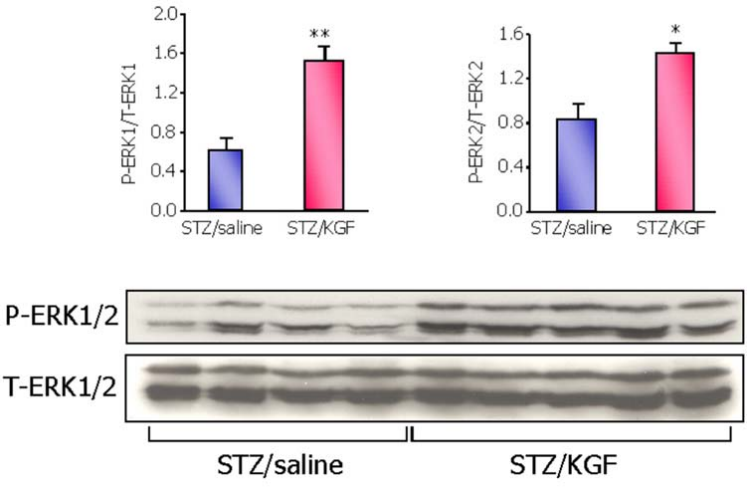
Activation of ERK1/2 pathway by KGF in the pancreas of neonatal diabetic rats. Two-day-old STZ diabetic rats were administrated with 3 mg/kg KGF, 20 minutes before the sacrifice. Pancreases were removed and pancreatic protein extracts were analyzed by western blot for the expression of P-ERK1/2 and total-ERK1/2 proteins. Four and five rats were analyzed in the STZ/saline and STZ/KGF group respectively.

We first investigated whether KGF induces ERK1/2 activation in the pancreas of neonatal diabetic rats. We found a significant increase of phosphorylated ERK1/2 in the whole pancreatic extracts of 2-day-old animals treated with KGF 20 minutes before the sacrifice (Figure 5).

#### Inactivation of ERK1/2 pathway by MEK1 inhibitors and its effects on KGF-induced duct cell proliferation

In order to investigate the signaling pathways involved in the transduction of the mitogenic effects of KGF on neonatal pancreatic duct cells showed previously [6], we silenced MEK1 protein expression by the use of anti-MEK1 morpholino-oligonucleotides, administrated in vivo to the diabetic pups. During the treatments, all pups tolerated standard morpholinos and MEK1-AS well. There were no adverse effects involving general appearance, body weight, and glycemia (data not show) and no pups died in relation with the administrated treatment.

**Figure 6:**
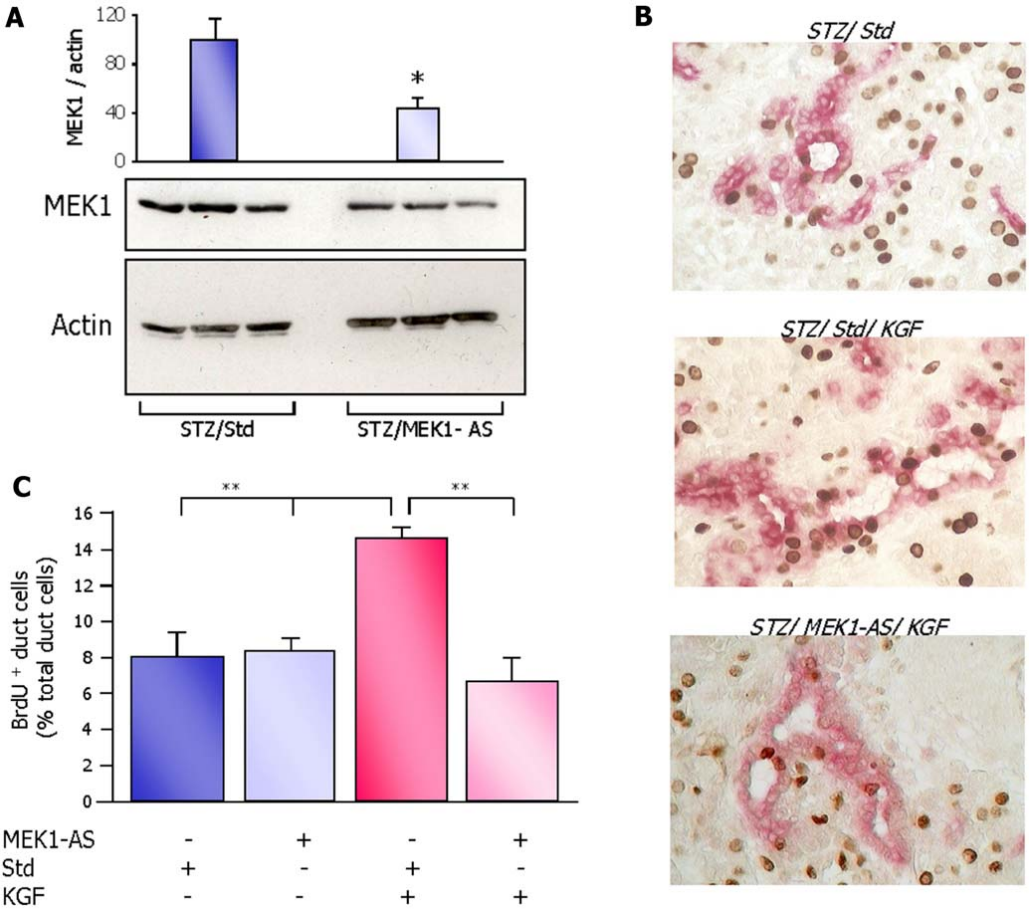
Inactivation of ERK1/2 pathway by MEK1-AS abolishes KGF-induced duct cell proliferation. (A) Rats from STZ/Std and STZ/MEK1-AS groups were sacrificed at day 2 post natal. Pancreases were removed and pancreatic protein extracts were analyzed by western blot using a specific MEK1 antibody. Representative blots and MEK1 protein quantification are shown in Figure 6A. Results are expressed as mean values ±sem. Four rats were analyzed in each experimental group. * *p*<0.05. (B) BrdU (brown nuclei) and CK20 staining (red cytosol) of pancreatic sections from 2-day-old STZ/Std/KGF and STZ/MEK1-AS/KGF newborns (actual magnification ×500). (C) Ductal cell proliferation was assessed by BrdU staining of pancreatic sections of STZ/Std, STZ/MEK1-AS, STZ/Std/KGF and STZ/MEK1-AS/KGF groups. BrdU labelling index of ductal cells in all experimental groups was evaluated in 2-day-old pups and the results were expressed as the percentage of BrdU-positive ductal cells. Three to five rats were analyzed in each experimental group. ** *p*<0.01.

Western blot analysis of whole pancreatic extracts from 2-day-old rats injected with MEK1-AS showed that the amount of MEK1 protein was significantly reduced (by 50%) in the pancreas, confirming the efficiency of morpholino-oligonucleotides to inhibit MEK1 translation in vivo (figure 6A). STZ/Std group exhibited no difference in pancreas morphology compared to STZ/saline group. Moreover duct cell proliferation assessed by BrdU incorporation method in STZ/Std group was similar to that of STZ/saline group (8.1±0.8% *vs* 7.6±0.3% respectively). Therefore for duct cell proliferation and differentiation studies, STZ/Std group has been taken as control for comparison with all other groups.

We showed that the inhibition of MEK1/ERK pathway dramatically reduced the proliferation of ductal cells induced by KGF in 2-day-old STZ rats as compared to STZ/Std group. These data indicate that the KGF-stimulated proliferation of neonatal duct cells is exclusively dependent on ERK1/2 activation (Figure 6B). We also found a decrease in the rate of proliferation of acinar cells (Figure 6B).

#### Activation of duct-to-beta cell differentiation by KGF

**Figure 7:**
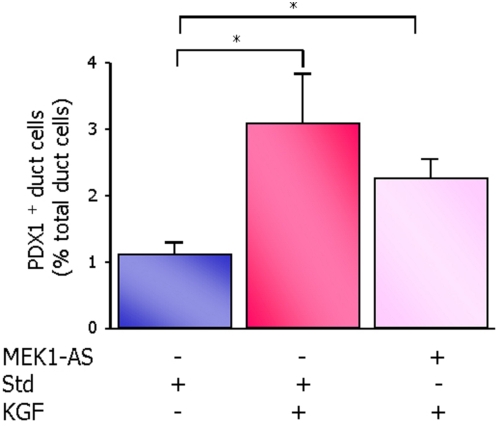
Activation of duct- to- beta cell differentiation by KGF. PDX1-positive/insulin-negative ductal cells were quantified after insulin/PDX1 double staining of pancreatic sections of the 2-day-old STZ/Std, STZ/Std/KGF and STZ/MEK1-AS/KGF rats. Results are expressed as the percentage of PDX1-positive/insulin-negative ductal cells over total ductal cells. Four rats were analyzed in each experimental group. * *p*<0.05.

In order to assess the mechanisms of ductal cell differentiation into beta cells in STZ rats, we analyzed the ductal expression of PDX1 and Glut 2, as intermediate steps in the process of beta cell neogenesis. We found that KGF significantly increased the number of PDX1-positive/insulin-negative ductal cells in the pancreas of 2-day-old pups (Figure 7). At the age of 4 days post natal, a slight but not significant increase in the number of PDX1-positive/insulin-negative ductal cells persisted in the KGF treated rats (not shown). PDX1 regulates the expression of a number of beta cell specific genes including Glut 2 [16], [17]. We sought to determine whether the early induction of PDX1 expression by KGF is further followed by the activation of downstream effectors involved in the acquisition of mature beta cell phenotype.

**Figure 8:**
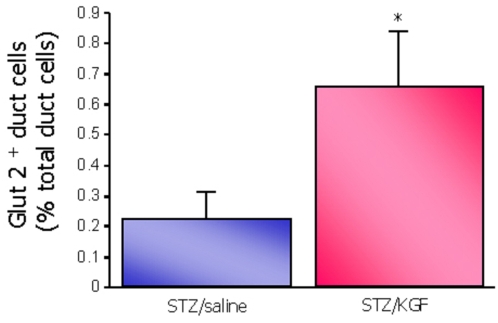
Activation of duct- to- beta cell differentiation by KGF. Glut 2-positive/insulin-negative cells were quantified after insulin/Glut 2 double staining of pancreatic sections of the 4-day-old STZ/saline and STZ/KGF groups. Results are expressed as the percentage of Glut 2-positive/insulin-negative ductal cells over total ductal cells. Four rats were analyzed in each experimental group. * *p*<0.05.

To address this point, we examined the expression of Glut 2, an important effector for glucose sensing in terminally differentiated beta cells, also reported as a marker of precursor cells with potential to differentiate into beta cells [18]. At the age of 2 days post natal (8 hours after KGF administration) the percentage of of Glut 2-positive/insulin negative ductal cells was not greater in the KGF treated group than that found in the age-matched untreated STZ group (not shown). At the age of 4 days post natal, the number of Glut 2-positive/insulin-negative ductal cells was significantly increased in the pancreases of rats treated with KGF compared to that found in the STZ control group (Figure 8).

Beta cell neogenesis can be indirectly estimated by the evaluation of the number of single or small clusters of insulin expressing cells within or in the vicinity of the epithelial wall of pancreatic ducts. At the age of 4 (0.61±0.17% of total ductal cells in the STZ group *vs* 1.62±0.07% of total ductal cells in the STZ/KGF group) and 7 days post natal (1.07±0.09% of total ductal cells in the STZ group *vs* 2.25±0.01% of total ductal cells in the STZ/KGF group), the number of single beta cells or clusters of beta cells budding from ducts was significantly higher (*p*<0.05) in the KGF treated groups compared to the age-matched untreated control groups.

#### Inactivation of ERK1/2 pathway and its impact on KGF-induced expression of PDX1 in ductal cells

Rats treated with MEK1-AS exhibited a slight but not significant decrease in the number of PDX1-positive ductal cells in response to 8 h KGF treatment as compared to STZ/Std rats treated with KGF (Figure 7) suggesting that ERK1/2 activation is not required for the expression of PDX1 in ductal cells.

#### Inactivation of PI3K/AKT pathway and its impact on KGF-induced expression of PDX1 in ductal cells

**Figure 9:**
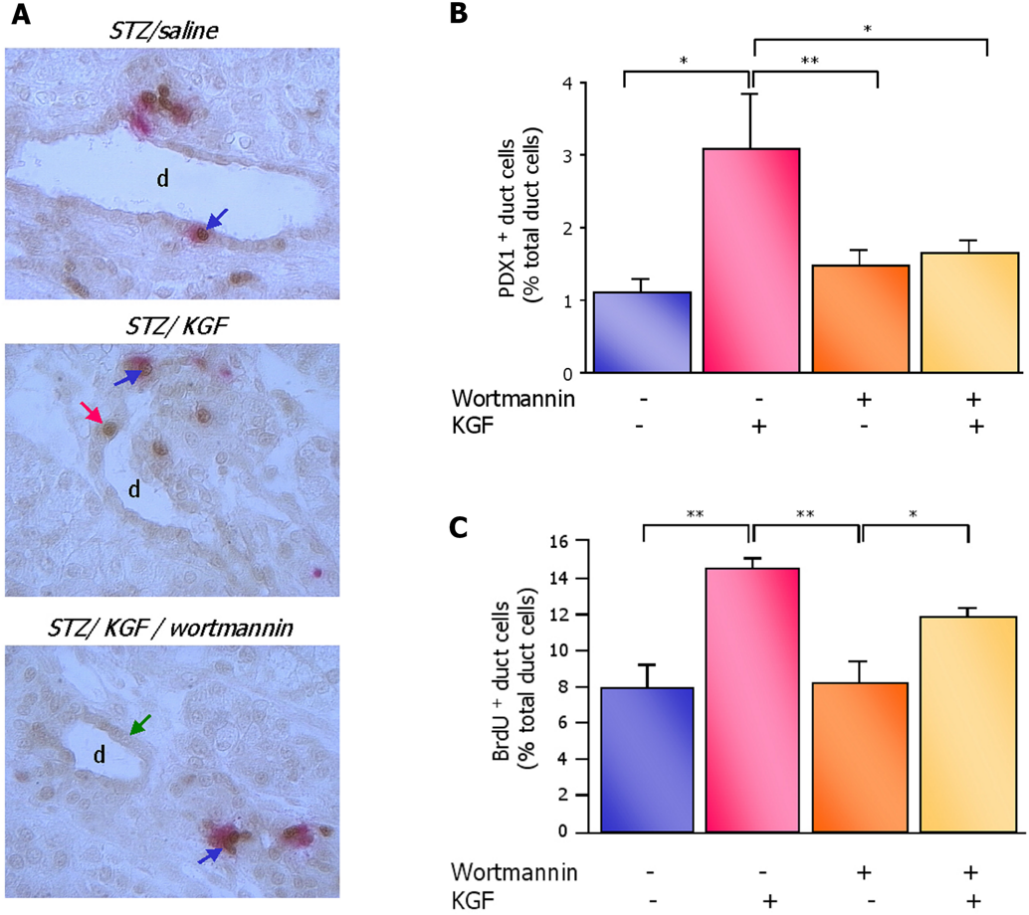
Inactivation of PI3K/AKT pathway by wortmannin abolishes KGF-induced duct-to-beta cell differentiation but does not alter the effect of KGF on proliferation. (A) Double staining for insulin (red cytosol) and PDX1 (brown nuclei) were performed on pancreatic sections of STZ, STZ/KGF, STZ/wortmannin and STZ/wortmannin/KGF rats (wortmannin was administrated at the dose of 0.75 mg/kg body weight). Differentiated beta cells express both insulin and PDX1 (blue arrow). A subset of ductal cells express PDX1 (red arrow) while the majority of ductal cells are PDX1 negative (green arrow) (actual magnification ×500). d = duct. (B) PDX1-positive / insulin-negative ductal cells were quantified after insulin/PDX1 staining on pancreatic sections of the 2-day-old STZ, STZ/KGF, STZ/wortmannin and STZ/wortmannin/KGF rats. Results are expressed as the percentage of PDX1-positive/insulin-negative cells over total ductal cells. Four rats were analyzed in each experimental group. * *p*<0.05; ** *p*<0.01. (C) Ductal cell proliferation was assessed by BrdU staining of pancreatic sections of the 2-day-old STZ, STZ/KGF, STZ/wortmannin and STZ/wortmannin/KGF rats and the results were expressed as the percentage of BrdU-positive ductal cells. Four rats were analyzed in each experimental group. * *p*<0.05; ** *p*<0.01.

The implication of the PI3K/AKT pathway in the induction of PDX1 expression in exocrine cells had been reported in an adult model of pancreatic regeneration [19]. In order to assess whether this signaling pathway is involved in the transduction of KGF signal in ductal cells in in the neonatal regenerating pancreas, we proceeded to the inactivation of PI3K by wortmannin. Treatment of rats with the PI3K inhibitor abolished the effect of KGF on PDX1 expression as evidenced by a significant decrease in the number of PDX1 positive ductal cells in the pancreas of 2-day-old KGF treated diabetic pups pre-treated with wortmannin, compared to the KGF treated group, while this treatment did not alter the basal number of PDX1 expressing ductal cells (Figures 9A and 9B).

#### Inactivation of PI3K/AKT pathway and its impact on KGF-induced ductal cell proliferation

We have also evaluated the rate of ductal cell proliferation in the STZ/wortmannin/KGF group, as compared to the STZ/KGF group. This parameter was found to be similar in both groups (Figure 9C), indicating that the PI3K/AKT pathway is implicated in the differentiation but not in the proliferation process in the pancreatic ductal cells. Interestingly, the proliferation of acinar cells was dramatically reduced in the pancreas of wortmannin treated rats, either alone or associated with the treatment with KGF (not shown).

## Discussion

Type 1 and type 2 diabetes are associated with absolute or relative loss of pancreatic beta cells. The shortage of islet tissue critically hampers the wide application of cell replacement therapy for type 1 diabetes and unfortunately patients with type 2 diabetes still suffer from the absence of curative treatments for the disease. Alternative cellular sources with potential to be induced to express beta cell phenotype can overcome beta cell deficiency and lead to major advances for the cure of this devastating disease. Pancreatic duct cells represent such an alternative source for generation of new beta cells [1] either ex-vivo prior to transplantation, or in vivo in the pancreas of diabetic patients.

During pancreas organogenesis, all pancreatic cell types arise from common precursor cells residing in ductal structures which result from evagination and branching of the ventral and dorsal pancreatic buds [20]–[22]. Ducts are also important for the generation of beta cells in the post natal life. This has been illustrated during the compensatory attempt to replace the normal beta cell population, for instance in animal models of cell injury induced by subtotal pancreatectomy [23]–[25] or in neonatal pancreas following beta cell destruction by the toxic agent streptozotocin [9], [26]. In these contexts apart from proliferation of existing beta cells, differentiation of precursor cells located in ductal epithelium participate to the recovery of the beta cell mass [9], [23], [27]–[33]. A substantial body of evidence in the literature thus designates pancreatic duct as an important source for generation of new beta cells both in vitro and in vivo [1], [25], [34]. Therefore, characterization of stimuli with potential to induce the expansion of ductal cells and/or to drive their differentiation into beta cells is of great importance.

We and others have previously showed that in vivo administration of KGF induces ductal cell proliferation [5] and beta cell regeneration in diabetic rats [6]. Modulation of signaling pathways by external stimuli represents key strategy used by the cellular machinery to regulate cell proliferation and differentiation processes. The understanding of these mechanisms is essential for the outcome of regenerative therapies of diabetes based on the induction duct cell proliferation and differentiation. The present study was undertaken to dissect the intracellular signaling pathways which mediate the growth and differentiation promoting effects of KGF in pancreatic ductal cells.

As a cell system to decipher the intracellular mechanisms underlying the effects of KGF on ductal cell proliferation in vitro, we used Panc-1 cells, a ductal cell line derived from human adenocarcinoma. This cell line has been used in several studies as a model for the induction of duct-to-beta cell differentiation [35], [36].

As these cells have high proliferative capacity in the presence of serum, all the experiments were conducted in the context of serum starvation in order to induce quiescence. We showed that KGF stimulated serum deprived Panc-1 cell proliferation as potently as did 10% FBS. The induction of cell proliferation by KGF in cultures of primary human ducts has been previously reported [34], [37]. However in these studies a thorough analysis of growth promoting potential of KGF and the signaling pathways involved, had not been made. The expression of specific KGF receptor (FGFR2IIIb) having been described in this cell line [38], [39], we sought to investigate the post-receptor signaling molecules which mediate the effects of KGF on cell proliferation. One of the most common signaling pathways linked to cell response to growth factors is the mitogen-activated protein kinases (MAPK) pathway. Among the three main MAPK cascades (ERK1/2, JNK and P38), ERK1/2 are predominantly activated by growth factors whereas P38 and JNK transduce stress-related stimuli such as heat shock and inflammatory cytokines [40], [41]. We analyzed the implication of ERK1/2 pathway in the mediation of KGF proliferative effects on ductal cells. The only known upstream activators of ERK1/2 are MEK proteins. We first inactivated MEK1 by its pharmacological inhibitor PD98059. We further confirmed the specificity of MEK inactivation by the use of antisense morpholino-oligonucleotides (MEK1-AS) specifically designed to inhibit MEK1 translation. Strikingly, in both cases blockade of ERK1/2 phosphorylation was associated with complete inhibition of proliferation induced by KGF. From these data we clearly established that ERK1/2 activation is necessary and sufficient for the mediation of growth promoting effects of KGF in pancreatic ductal cells in vitro. The mediation of mitogenic effects of KGF by ERK1/2 signaling has also been reported in other cell types such as endometrial cells [42], [43] while other effects of KGF such as regulation of cell migration were mediated by MAP Kinase P38 [43]. The cell cycle is regulated at various checkpoints, of which the G1–S phase represents the most important step. At this checkpoint, extra-and intracellular signals are integrated and transmitted into cells to determine whether they enter the cell division phase, apoptosis or the quiescence G0 phase [44]. Cyclins are major regulators of cell cycle transition and by their binding to cyclin-depedent kinases they act as molecular switches for the G0/G1-S phase transition in many cell types. We first established that cyclin D1 was expressed in Panc-1 cells while cyclin D2 was not detectable in this cell type. KGF treatment of Panc-1 cells significantly increased the levels of cyclin D1 protein and the blockade of the ERK signaling by PD98059 prevented the induction of cyclin D1 expression by KGF. Kornmann et al. have previously showed that cyclin D1 is over-expressed in ductal cancer cells and treatment of cells with cyclin D1 antisense was associated with significant inhibition of in vitro duct cell proliferation [45]. However the induction of cyclin D1 by KGF in pancreatic ductal cells has to date not been addressed. Thus, our report in support of previous studies [45], [46] suggests that cyclin D1 may represent a final common effector for mitogenic signaling via KGF receptor.

Although previous in vitro studies have partly identified the signaling pathways of growth factor-mediated proliferation in pancreatic ductal cells in vitro [37], [47], the intracellular mechanisms of KGF-induced duct cell proliferation in vivo are still poorly understood. Therefore, in this study we explored how the pathways induced by KGF in vitro, translate into their in vivo activation. In a previous work we have reported that in vivo administration of KGF to neonatal diabetic rats stimulated beta cell regeneration via the induction of duct cell proliferation and their subsequent differentiation into insulin producing cells [6]. Here we confirmed the in vivo growth promoting effect of ductal cells by KGF in the pancreas of STZ diabetic rats. To determine the relevance of our in vitro findings to the in vivo situation we assessed the implication of ERK1/2 pathway in the KGF-induced duct cell proliferation. We used a specific approach based on the administration of anti-MEK1 antisense oligonucleotides to the pups. We demonstrated for the first time that MEK1 can be successfully knocked down in the pancreas by the use of antisense-oligonucleotides in vivo. This approach, more specific than pharmacological inhibition and less harmful than adenovirus mediated gene inactivation was well tolerated by the pups and no general deleterious effects due to the downregulation MEK1 in other organs during the time course of our study were observed (not shown). This is the first demonstration of the in vivo use of morpholino-oligonucleotides as a safe and specific post-transcriptional gene silencing approach in newborn rats. The blockade of ERK1/2 in vivo totally abolished the KGF-induced proliferation of ductal cells indicating that, at least in the neonatal pancreas, ERK1/2 pathway is the exclusive signaling pathway responsible for duct cell proliferation induced by KGF. The mediation of growth promoting effects of KGF by ERK1/2 signaling has also been reported in culture of embryonic pancreatic rudiments [47]. ERK1/2 activation has been shown to occur in a model of adult pancreas regeneration after pancreatectomy [48]. Immunohistochemical analysis revealed that in this model, ERK phosphorylation can be seen only in duct cells and not in acinar cells during pancreatic regeneration [49], [50]. Ductal compartment of the pancreas is admitted to harbour precursor cells for islet neogenesis. Therefore, the activation of ERK proteins in the duct in regenerating pancreas may play an indirect role in endocrine cell neogenesis by increasing the number of so-called progenitor cells. Here we have further explored the possibility that, beyond the stimulation of cell proliferation in the ductal compartment, other steps of beta cell neogenesis from ducts could be directly modulated by KGF. In order to address this question, we examined the expression of beta cell specific markers in ductal cells in the pancreas of neonatal diabetic rats. The homeodomain protein PDX1 is a prominent transcription factor for pancreas morphogenesis during development [20], [51], [52] and a transcriptional regulator of insulin synthesis and secretion in mature beta cells [53]–[55]. It has been strongly suggested that during the postnatal life, PDX1 may also be essential for neogenesis of endocrine cells by differentiation from precursor cells that reside among the cells lining the pancreatic ducts [56]–[58]. The examination of pancreatic sections revealed that the number of PDX1-positive/insulin-negative cells was significantly higher among ductal cells of KGF treated STZ rats. PDX1-positive/insulin-negative cells can be considered as cells committed toward a beta cell-like phenotype without having achieved terminal differentiation yet. This novel finding designates for the first time KGF as a differentiation inducing agent through the stimulation of PDX1 expression in ductal cells. In a study in primary islets and in INS1 beta cells, ERK1/2 activation has been shown to be required for the regulation of important transcription factors such as Beta2, PDX-1, and E12/47 [59]. Therefore we assessed the implication of ERK1/2 pathway in the induction of PDX1 expression in ductal cells induced by KGF. We found that the blockade of this pathway by the administration of anti-MEK1 antisense-oligonucleotides did not affect the KGF-induced expression of PDX1 in ducts, suggesting that the induction of PDX1 in those cells in not dependent upon ERK1/2 activation. Several papers by Evers group have reported that in the context of pancreas regeneration after pancreatectomy in adult rodents, the induction of PDX1 expression in duct cells is regulated by the PI3K pathway [49], [50]. However whether KGF acts through this pathway to induce PDX1 expression in ductal cells had not been reported. We addressed this question in the present study and showed that the effect of KGF on the ductal expression of PDX1 is totally abolished in animals treated with wortmannin, providing evidence on the role of the PI3K/AKT pathway in the mediation of the KGF signal for PDX1 expression in ducts. As an additional hallmark of the progression of duct-to-beta cell differentiation process, we investigated the expression of Glut 2 in ductal cells. Glut 2, a transcriptional target of PDX1, is an essential effector of the glucose sensing machinery in terminally differentiated beta cells. However, Glut 2 has also been reported as a marker of precursor cells during the early stages of pancreas development [60] and in a subset of precursor cells during beta cell regeneration in experimental models of diabetes [18]. Our histological examination of the pancreatic sections revealed that the number of Glut 2-positive/insulin-negative cells among the ductal compartment is significantly increased in rats treated with KGF, suggesting that this growth factor induces duct-to-beta cell differentiation by the sequential activation of beta cell specific markers in ductal cells. Whether the expression of Glut 2 in ductal cells is induced by KGF directly or by transactivation effect of PDX1 is not clear but the fact that at an earlier stage (day 2 post natal) the number of Glut 2-positive ductal cells was not greater than that found in the age-matched untreated control group, suggests that the increased number of Glut 2-positive ductal cells observed at day 4 post natal might be due to the induction of Glut 2 by PDX1.

Although PDX1 and Glut 2 are required for the initiation of the process of beta cell differentiation, the expression of many other factors including transcription factors are necessary for the full differentiation of beta cells [61]. However, only the presence of insulin can testify that the process of beta cell neogenesis has reached full completion. Therefore we evaluated the number of single or small clusters of insulin containing cells within or in the vicinity of the ductal epithelium.

The previous [6] and the present observation that the number of duct-associated insulin-positive cells was greater in KGF treated rats at the age of 4 and 7 days post natal compared to the age-matched untreated rats suggests that ductal cells expressing PDX1 and Glut 2 eventually differentiate into mature insulin producing beta cells.

To the best of our knowledge, our study is the first in providing evidence of a direct differentiation promoting effect of KGF on ductal cells and for the identification of the signaling pathways involved in this process. We demonstrated that in vivo, in the context of beta cell deficiency, exogenous KGF acts on pancreatic ductal cells through two distinct mechanisms: 1) via the induction of their proliferation through an ERK1/2 dependent mechanism. The activation of cell proliferation in ductal compartment which includes that of progenitor cells would increase the number of the latter and indirectly promote the process of beta cell neogenesis. 2) via the induction of PDX1 expression through a PI3K dependent mechanism in a subpopulation of ductal cells, committing those cells towards differentiation into beta cell. Different duct cell subpopulations may differ in their precursor capacity. We have demonstrated that KGF acts in a unique cell type, via distinct signaling pathways to trigger the commitment of different subsets of ductal cells towards a beta cell-like phenotype. The transition from duct to beta cell phenotype requires a complex network of signaling pathways whose activation / inactivation in a finely coordinated manner ensures the proper differentiation process to take place [35]. The identification of agents with the potential to trigger this transition is of great interest. Our present work designates KGF as an effective agent for the in vivo induction of duct-to-beta cell differentiation and might have have important implications for the regenerative therapies of diabetes.
